# Supplementary material for: From sequence to enzyme mechanism using multi-label machine learning
Source: BMC Bioinformatics. 2014 May 19;15:150. doi: 10.1186/1471-2105-15-150 (PMC4229970; doi:10.1186/1471-2105-15-150)
Supplement: Additional file 2 — Java code of ml2db. Additional file ml2db_code.tar.gz contains the Java source code to run the multi-label machine learning experiments and save the results to database. The code’s Javadoc is included. [file 1471-2105-15-150-S2.zip › additional file 2/ml2db/utils/doc/uk/ac/standrews/utils/test/Data.html]

Data


JavaScript is disabled on your browser.


- Overview
- Package
- Class
- Use
- Tree
- Deprecated
- Index
- Help

- Prev Class
- Next Class

- Frames
- No Frames

- All Classes

- Summary:
- Nested |
- Field |
- Constr |
- Method

- Detail:
- Field |
- Constr |
- Method


uk.ac.standrews.utils.test

## Class Data

- java.lang.Object
- - junit.framework.Assert
  - - junit.framework.TestCase
    - - uk.ac.standrews.utils.test.Data

- All Implemented Interfaces:
  :   junit.framework.Test

  ---

    

  ```
  public class Data
  extends junit.framework.TestCase
  ```

  Class

  Version:
  :   3 Jun 2008

  Author:
  :   Luna De Ferrari luna.deferrari-at-ed.ac.uk

- - ### Field Summary

    Fields

    | Modifier and Type | Field and Description |
    | --- | --- |
    | `static java.lang.String` | `KEY1` |
    | `static java.lang.String` | `KEY2` |
    | `static java.lang.String` | `KEY3` |
    | `static java.lang.String` | `KEYA` |
    | `static java.lang.String` | `KEYB` |
    | `static java.lang.String` | `KEYC` |
    | `static java.lang.String` | `VAL1` |
    | `static java.lang.String` | `VAL2` |
    | `static java.lang.String` | `VAL3` |
    | `static java.lang.String` | `VALA` |
    | `static java.lang.String` | `VALB` |
  - ### Constructor Summary

    Constructors

    Constructor and Description

    `Data()`
  - ### Method Summary

    Methods

    | Modifier and Type | Method and Description |
    | --- | --- |
    | `static java.util.Vector<java.lang.String>` | `emptyKeys()` |
    | `static java.util.Vector<java.lang.String>` | `emptyValue()` |
    | `static java.util.Vector<java.lang.String>` | `externalKeys()` |
    | `static java.util.Vector<java.lang.String>` | `internalKeys()` |
    | `static java.util.Vector<java.lang.String>` | `internalNumericalValues()` |
    | `static java.util.Vector<java.lang.String>` | `internalOnesAndEmptyStringValues()` |
    | `static java.util.Vector<java.lang.String>` | `internalValues()` |
    | `static java.util.Vector<java.lang.String>` | `keys()` |
    | `static java.util.Vector<java.lang.String>` | `keys2()` |
    | `static java.util.Vector<java.lang.String>` | `values()` |
    | `static java.util.Vector<java.lang.String>` | `values2()` |

    - ### Methods inherited from class junit.framework.TestCase

      `countTestCases, getName, run, run, runBare, setName, toString`
    - ### Methods inherited from class junit.framework.Assert

      `assertEquals, assertEquals, assertEquals, assertEquals, assertEquals, assertEquals, assertEquals, assertEquals, assertEquals, assertEquals, assertEquals, assertEquals, assertEquals, assertEquals, assertEquals, assertEquals, assertEquals, assertEquals, assertEquals, assertEquals, assertFalse, assertFalse, assertNotNull, assertNotNull, assertNotSame, assertNotSame, assertNull, assertNull, assertSame, assertSame, assertTrue, assertTrue, fail, fail, failNotEquals, failNotSame, failSame, format`
    - ### Methods inherited from class java.lang.Object

      `equals, getClass, hashCode, notify, notifyAll, wait, wait, wait`

- - ### Field Detail


    - #### KEY1

      ```
      public static final java.lang.String KEY1
      ```

      See Also:
      :   Constant Field Values


    - #### KEY2

      ```
      public static final java.lang.String KEY2
      ```

      See Also:
      :   Constant Field Values


    - #### KEY3

      ```
      public static final java.lang.String KEY3
      ```

      See Also:
      :   Constant Field Values


    - #### KEYA

      ```
      public static final java.lang.String KEYA
      ```

      See Also:
      :   Constant Field Values


    - #### KEYB

      ```
      public static final java.lang.String KEYB
      ```

      See Also:
      :   Constant Field Values


    - #### KEYC

      ```
      public static final java.lang.String KEYC
      ```

      See Also:
      :   Constant Field Values


    - #### VAL1

      ```
      public static final java.lang.String VAL1
      ```

      See Also:
      :   Constant Field Values


    - #### VAL2

      ```
      public static final java.lang.String VAL2
      ```

      See Also:
      :   Constant Field Values


    - #### VAL3

      ```
      public static final java.lang.String VAL3
      ```

      See Also:
      :   Constant Field Values


    - #### VALA

      ```
      public static final java.lang.String VALA
      ```

      See Also:
      :   Constant Field Values


    - #### VALB

      ```
      public static final java.lang.String VALB
      ```

      See Also:
      :   Constant Field Values
  - ### Constructor Detail


    - #### Data

      ```
      public Data()
      ```
  - ### Method Detail


    - #### emptyKeys

      ```
      public static java.util.Vector<java.lang.String> emptyKeys()
      ```


    - #### emptyValue

      ```
      public static java.util.Vector<java.lang.String> emptyValue()
      ```


    - #### externalKeys

      ```
      public static java.util.Vector<java.lang.String> externalKeys()
      ```


    - #### internalKeys

      ```
      public static java.util.Vector<java.lang.String> internalKeys()
      ```


    - #### internalNumericalValues

      ```
      public static java.util.Vector<java.lang.String> internalNumericalValues()
      ```


    - #### internalOnesAndEmptyStringValues

      ```
      public static java.util.Vector<java.lang.String> internalOnesAndEmptyStringValues()
      ```


    - #### internalValues

      ```
      public static java.util.Vector<java.lang.String> internalValues()
      ```


    - #### keys

      ```
      public static java.util.Vector<java.lang.String> keys()
      ```


    - #### keys2

      ```
      public static java.util.Vector<java.lang.String> keys2()
      ```


    - #### values

      ```
      public static java.util.Vector<java.lang.String> values()
      ```


    - #### values2

      ```
      public static java.util.Vector<java.lang.String> values2()
      ```


- Overview
- Package
- Class
- Use
- Tree
- Deprecated
- Index
- Help

- Prev Class
- Next Class

- Frames
- No Frames

- All Classes

- Summary:
- Nested |
- Field |
- Constr |
- Method

- Detail:
- Field |
- Constr |
- Method
